# Early nucleolar responses differentiate mechanisms of cell death induced by oxaliplatin and cisplatin

**DOI:** 10.1016/j.jbc.2021.100633

**Published:** 2021-04-03

**Authors:** Emily C. Sutton, Victoria J. DeRose

**Affiliations:** 1 Department of Biology, University of Oregon, Eugene, Oregon, USA; 2 Institute of Molecular Biology, University of Oregon, Eugene, Oregon, USA; 3 Department of Chemistry and Biochemistry, University of Oregon, Eugene, Oregon, USA

**Keywords:** nucleolus, anticancer drug, ribosomal RNA, stress response, DNA damage response, pre-rRNA, ribosome biogenesis, nucleolar stress, ActD, actinomycin D, CV, coefficient of variation, DACH, diaminocyclohexane, DDR, DNA damage response, DMEM, Dulbecco's modified Eagle medium, FBS, fetal bovine serum, HMGB, high mobility group box protein, NPM1, nucleolar protein nucleophosmin, Pol I, Polymerase I, rDNA, ribosomal DNA, rRNA, ribosomal RNA, UBF, upstream binding factor

## Abstract

Recent reports provide evidence that the platinum chemotherapeutic oxaliplatin causes cell death *via* ribosome biogenesis stress, while cisplatin causes cell death *via* the DNA damage response (DDR). Underlying differences in mechanisms that might initiate disparate routes to cell death by these two broadly used platinum compounds have not yet been carefully explored. Additionally, prior studies had demonstrated that cisplatin can also inhibit ribosome biogenesis. Therefore, we sought to directly compare the initial influences of oxaliplatin and cisplatin on nucleolar processes and on the DDR. Using pulse-chase experiments, we found that at equivalent doses, oxaliplatin but not cisplatin significantly inhibited ribosomal RNA (rRNA) synthesis by Pol I, but neither compound affected rRNA processing. Inhibition of rRNA synthesis occurred as early as 90 min after oxaliplatin treatment in A549 cells, concurrent with the initial redistribution of the nucleolar protein nucleophosmin (NPM1). We observed that the nucleolar protein fibrillarin began to redistribute by 6 h after oxaliplatin treatment and formed canonical nucleolar caps by 24 h. In cisplatin-treated cells, DNA damage, as measured by γH2AX immunofluorescence, was more extensive, whereas nucleolar organization was unaffected. Taken together, our results demonstrate that oxaliplatin causes early nucleolar disruption *via* inhibition of rRNA synthesis accompanied by NPM1 relocalization and subsequently causes extensive nucleolar reorganization, while cisplatin causes early DNA damage without significant nucleolar disruption. These data support a model in which, at clinically relevant doses, cisplatin kills cells *via* the canonical DDR, and oxaliplatin kills cells *via* ribosome biogenesis stress, specifically *via* rapid inhibition of rRNA synthesis.

The nucleolus is the site of ribosome biogenesis, a process that includes transcription of ribosomal RNA (rRNA) by RNA Polymerase I (Pol I), processing of rRNA, and assembly of ribosomal subunits. Nucleolar morphology can be used as a prognostic factor for tumor severity, and nucleolar size is correlated to node and receptor status in breast cancer as well as length of disease-free survival period (1). Functionally, ribosome biogenesis is closely associated with cellular processes such as the cell cycle, further linking the dysregulation of this process to cancer and other diseases (2). While upregulated ribosome biogenesis is associated with cancer, inhibition of ribosome biogenesis can lead to activation of the tumor suppressor protein p53 *via* the nucleolar stress response. This has made ribosome biogenesis and the nucleolus desirable targets for potential chemotherapeutic agents (3, 4, 5).

A few small molecules induce a cytotoxic nucleolar stress response by selectively inhibiting rRNA synthesis by Pol I. Actinomycin D (ActD) is known to selectively inhibit Pol I at low doses, an effect attributed to its propensity to target GC-rich regions of DNA, including ribosomal DNA (rDNA) (6). It is used effectively to treat specific types of tumors, but its clinical efficacy has been limited in scope (2). BMH-21 was identified in a screen for compounds that induce a p53 response (7) and later found to inhibit Pol I (6). CX-5461 was identified in a screen for selective Pol I inhibitors (8). The ability of BMH-21 to inhibit rRNA transcription is currently believed to be due to stalling of Pol I transcription followed by degradation of the Pol I subunit RPA194 (6, 9). The mechanism of CX-5461 remains more elusive, with recent evidence suggesting that it may primarily exert its cytotoxic effects by way of topoisomerase II inhibition rather than Pol I inhibition as previously believed (10, 11).

Platinum anticancer compounds have also been shown to disrupt nucleolar function. Until relatively recently, it was believed that all platinum-based chemotherapeutic agents, including cisplatin and oxaliplatin (Fig. 1*A*), exerted their cytotoxic effects by triggering the DNA damage response (DDR) (12, 13). In 2017, Bruno *et al.* (14) used an RNAi screening approach to identify ribosome biogenesis inhibition as a primary mechanism of oxaliplatin cytotoxicity in cancer cells, whereas cisplatin acts *via* the canonical DDR. This distinction has been supported by later studies demonstrating more extensive redistribution of the protein nucleophosmin (NPM1) from the nucleolus to the nucleoplasm—a hallmark of the nucleolar stress response—upon treatment with oxaliplatin and derivates (15). Because prior studies have suggested a more complex relationship between nucleolar stress processes and these platinum drugs, further investigation into the differences between cisplatin and oxaliplatin with regard to their ability to disrupt nucleolar processes is warranted.

**Figure 1:**
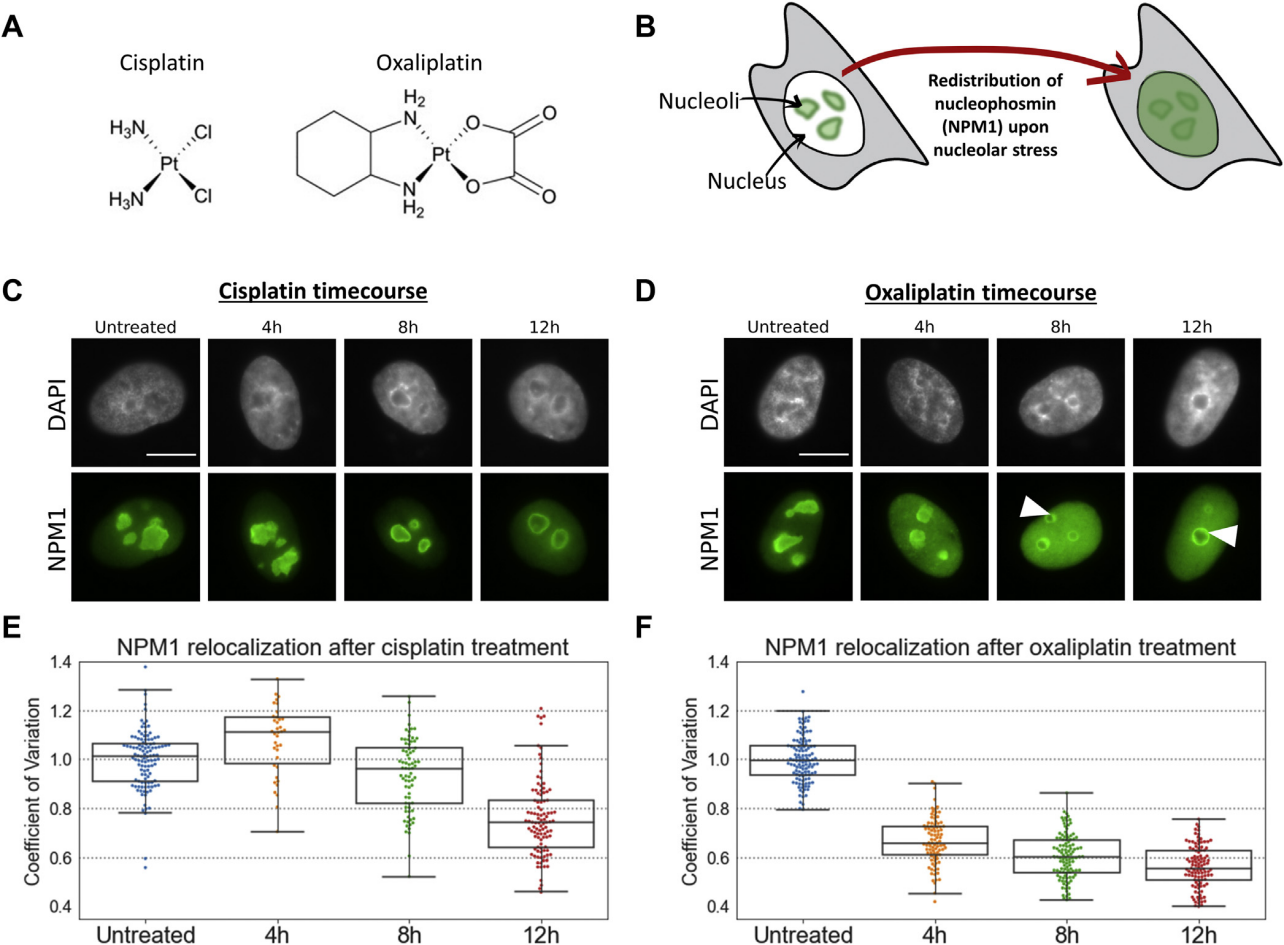
**Redistribution of NPM1 upon treatment with platinum compounds.***A*, structures of cisplatin and oxaliplatin. *B*, schematic showing the redistribution of NPM1 from the periphery of the nucleolus to the nucleoplasm, a hallmark of the nucleolar stress response. *C*, immunostaining of NPM1 (*green*) in A549 cells at 4, 8, and 12 h after treatment with 10 μM cisplatin. DAPI staining (*gray*) shows the nuclear DNA. *D*, immunostaining of NPM1 in A549 cells at 4, 8, and 12 h after treatment with 10 μM oxaliplatin. Cap-like structures indicated with *arrows*. *E*, quantification of NPM1 relocalization after cisplatin treatment. *F*, quantification of NPM1 relocalization after oxaliplatin treatment. A lower coefficient of variation (CV) indicates more extensive NPM1 redistribution and therefore more robust nucleolar stress. CV calculations, and boxplot presentation as described in the methods. For each treatment data set, *boxes* represent median, first, and third quartiles, and *vertical lines* are the range of data with outliers defined in the methods. Statistical significance testing at 4 h of treatment across three separate testing days is shown in Fig. S3. All scale bars are 10 μm.

While no studies to date have fully characterized how oxaliplatin might induce ribosome biogenesis stress, its ability to do so is documented in existing literature. Robust oxaliplatin-induced redistribution of NPM1 has been demonstrated (14, 15, 16, 17), and it has been shown to inhibit rRNA transcription and induce rearrangement of other nucleolar proteins at low doses (6.25 μM) in human fibrosarcoma cells (17). Proteomics studies have shown changes in levels of proteins related to ribosome biogenesis upon oxaliplatin treatment (18). However, in that study it was concluded that nucleolar stress was a consequence of DNA damage.

The relationship between nucleolar stress induction and cisplatin is more complex. While there is evidence for cisplatin’s ability to affect ribosome biogenesis and other nucleolar processes, recent data and an evaluation of existing literature suggest that these observed mechanisms may not be clinically relevant. Some examinations of cisplatin treatment in HeLa cells have demonstrated a reduction in rRNA synthesis by transcription run-on assay, along with redistribution of Pol I and Upstream Binding Factor (UBF), a transcription factor for Pol I (19). Morphological changes in the nucleolus have been observed at early stages of cell death induced by high doses (40 μM) of cisplatin (20). Reduction of transcription follows colocalization of Pol I and coilin 6 h after cisplatin treatment in HeLa cells, and this effect on transcription can be reversed by siRNA silencing of coilin (21). Hamdane *et al.* (22) have shown that 30 μM of cisplatin treatment leads to displacement of UBF from rDNA and inhibition of rRNA transcription in mouse embryonic fibroblasts, and high doses (50–100 μM) have been shown to inhibit rRNA transcription and induce nucleolar disruption in human fibrosarcoma cells (17).

Cisplatin does not induce the same robust level of NPM1 translocation as oxaliplatin does (14, 15). In sum, there is a clear difference between the ability of these highly similar platinum compounds to affect nucleolar processes. An overarching goal of research into these compounds is to better understand the mechanisms behind their differential efficacy and resistance profiles, and recent evidence suggests that it is decreasingly likely that any effects of cisplatin on nucleolar processes bear relevance to its clinical applications, with the inverse being true of oxaliplatin.

Further exploration of the mechanisms of action of these drugs and an examination of their ability to induce nucleolar stress are warranted. Previously, our lab has demonstrated some structural constraints for Pt(II) drugs to cause stress. The diaminocyclohexane (DACH) ligand of oxaliplatin proved critical, and while some changes to its size and aromaticity could be tolerated, compounds with different ring orientations did not induce stress (15). This proved valuable in understanding what types of Pt(II) molecules can lead to this response, but the biological mechanism by which oxaliplatin-like compounds induce nucleolar stress and cause NPM1 redistribution is still not well understood. For example, Pt(II) compounds are known to cause DNA damage, which can cause nucleolar stress indirectly by inhibiting Pol I (23). This relationship between DNA damage and nucleolar stress has yet to be examined in the specific context of Pt(II)-induced nucleolar stress. Some previous work has closely examined the ability of oxaliplatin and cisplatin, among other chemotherapeutic compounds, to inhibit rRNA synthesis and processing as well as rearrangement of specific nucleolar proteins (17). However, these studies examined cisplatin’s nucleolar effects at doses likely above clinical relevance and did not directly link this Pt(II)-induced inhibition to p53 stabilization after treatment with relevant Pt(II) doses. We sought to build on this work by utilizing similar techniques at time points and doses relevant to these critical processes. This paper marks a step forward in understanding which nucleolar processes do and do not cause Pt(II)-induced nucleolar stress by examining rRNA synthesis and processing alongside NPM1 redistribution at relevant time points, assessing DNA damage, and further characterizing protein redistribution behavior in Pt(II) stress conditions. We find that rRNA transcription is inhibited at early time points by oxaliplatin, but not cisplatin, and that this inhibition correlates with onset of NPM1 redistribution and precedes the formation of canonical fibrillarin-containing nucleolar caps. We also conclude that rRNA transcription inhibition is not preceded by a DDR during oxaliplatin treatment and that previous observations of cisplatin-induced nucleolar stress are likely reflecting events downstream of DDR.

## Results

### Onset of NPM1 redistribution

The protein NPM1 normally resides mainly in the periphery of the nucleolus and its relocalization to the nucleoplasm is a hallmark of the nucleolar stress response (Fig. 1*B*). It has previously been shown that both cisplatin and oxaliplatin can cause redistribution of NPM1 but with much higher doses of cisplatin (100 μM) than oxaliplatin (6.25 μM) in the same cell lines (17). Additionally, there has been little exploration of the molecular events preceding NPM1 redistribution upon drug treatment. We have previously measured NPM1 redistribution after 24 h of treatment with Pt(II) compounds (15). In order to directly compare cisplatin and oxaliplatin and to initially bracket a time course for onset of NPM1 distribution, A549 cells were treated for 4, 8, and 12 h with 10 μM cisplatin or oxaliplatin. A treatment concentration of 10 μM was chosen because it is a sufficient concentration to induce nucleolar stress without killing a large portion of the cellular population based on our previous findings (15). Cells were then fixed, permeabilized, and immunostained for NPM1 (Fig. 1, *C* and *D*). The extent of NPM1 redistribution was calculated as previously described (15, 16) by determining the coefficient of variation (CV) of NPM1 pixel intensities within each nucleus. These CV values were then normalized to the average CV for an untreated control and plotted by treatment group (Fig. 1, *E* and *F*). A lower CV value indicates more broadly distributed NPM1 in the nucleoplasm and more extensive nucleolar stress.

We determined that significant NPM1 translocation from the nucleolus is observed by 4 h of treatment with oxaliplatin and that the translocation becomes more extensive with time (Fig. 1, *D* and *F*, Fig. S3). By contrast, at the same treatment concentration and time period, only a minor amount of NPM1 translocation occurs upon cisplatin treatment (Fig. 1, *C* and *E*). After oxaliplatin treatment, we observed rounding of the nucleoli as previously described (15). Also of note is the appearance of bulging regions of NPM1 around the periphery of many of the rounded nucleoli after oxaliplatin treatment (Fig. 1*D*, white arrows). These features resemble “nucleolar caps” or structures that have been reported to form after exposure to ribosomal DNA damage and other nucleolar stressors (17, 24). Interestingly, NPM1 is not among the proteins previously reported to comprise nucleolar caps, with some studies expressly stating that NPM1 does not compartmentalize into nucleolar caps (24, 25). This rapid observable response with oxaliplatin treatment that includes NPM1 redistribution, morphological features such as nucleolar rounding, and the appearance of putative cap-like structures all raise interesting questions about the molecular processes behind them.

### Oxaliplatin but not cisplatin causes inhibition of rRNA transcription

NPM1 redistribution is just one marker of nucleolar stress, and while it is a robust and ubiquitous marker, observation of this redistribution with accompanying morphological changes does not address the molecular mechanisms by which Pt(II) compounds might be inducing nucleolar stress. Several known triggers of nucleolar stress include inhibition of any of the stages of ribosome biogenesis, DNA damage, or direct perturbation of nucleolar structure (26, 27, 28). We sought to narrow down the potential causes of oxaliplatin-induced NPM1 redistribution by examining three processes known to induce nucleolar stress—inhibition of rRNA synthesis, inhibition of rRNA processing, and DNA damage.

It has been established that inhibition of rRNA synthesis and processing are linked to nucleolar stress and NPM1 redistribution (28). Prior studies on the influences of cisplatin and oxaliplatin on rRNA synthesis have yielded mixed results. Using pulse-chase approaches, it has been shown that both cisplatin and oxaliplatin can inhibit rRNA transcription but that neither affects rRNA processing. However, in these studies, cisplatin causes inhibition between 40 and 100 μM, doses above the IC-50 values in the cell lines used and also thought to be above clinical relevance (17, 22). Other studies have shown stark differences between cisplatin and oxaliplatin with regard to their ability to affect rRNA transcription. Using RT-qPCR, Bruno *et al.* observed decreases in pre-rRNA levels after 30 min of treatment with oxaliplatin and ActD, followed by marked increases between 1 and 6 h of continuous treatment (14). Treatment with cisplatin did not demonstrate this trend in pre-rRNA levels. These data were used to support the conclusion that oxaliplatin acts as a ribosome biogenesis inhibitor like ActD, while cisplatin works by a different mechanism (namely DNA damage).

**Figure 2:**
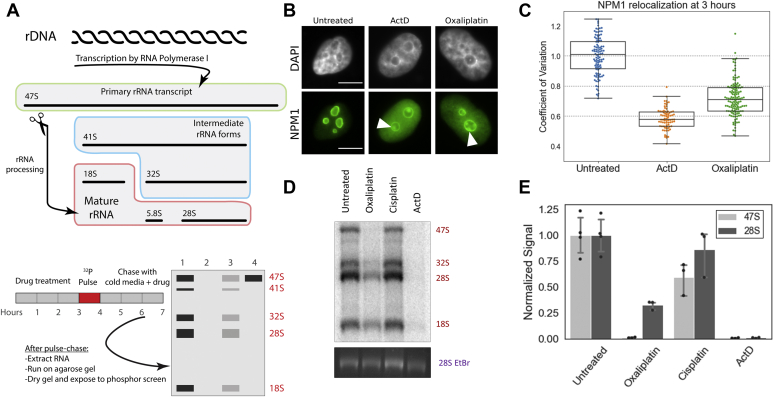
**NPM1 relocalization and inhibition of rRNA transcription in A549 cells after 3 h of treatment with platinum compounds.***A*, schematic of rRNA processing and pulse-chase experiment. *B*, NPM1 relocalization after 3 h of treatment with 10 μM oxaliplatin and with positive control ActD (5 nM). Scale bars are 10 μm. Cap-like structures indicated by *arrows*. *C*, quantification of NPM1 relocalization 3 h after treatment with 10 μM oxaliplatin or the positive nucleolar control of 5 nM ActD. *D*, results of pulse-chase experiment. Cells were treated with 10 μM of cisplatin or oxaliplatin or 5 nM of ActD for 3 h prior the pulse step. *Bottom frame* shows total RNA (EtBr stain of 28S rRNA) while *top image* shows ^32^P labeled rRNA. Transcript sizes are shown on the *right*. *E*, quantification of pulse-chase data from the gel image in 2D. Error bars represent the standard deviation of three replicates separately treated in three wells of A549 cells across 2 days.

To clarify the influence of cisplatin and oxaliplatin on rRNA transcription and processing and to determine the relationship of NPM1 redistribution to these effects, we conducted pulse-chase radiolabeling experiments using a method previously described (Fig. 2*A*) (17). After treatment of A549 cells with compounds for a fixed period, cells were incubated in Pt(II)-free media containing ^32^P-labeled phosphate, which would be incorporated into any newly synthesized RNA in the “pulse” step. Media was then replaced with cold, Pt(II)-containing media to track the processing fate of any newly synthesized RNA. Both the ^32^P-labeled and total RNA were visualized. Low-dose (5 nM) ActD is used as a positive control for transcription inhibition. NPM1 relocalization was measured at time points correlating to the beginning of the pulse step.

At 3 h of drug treatment, robust NPM1 relocalization is observed for both oxaliplatin and the positive control of ActD (Fig. 2, *B* and *C*). In addition, nucleolar rounding and cap-like structures containing NPM1 are observed following treatment with both oxaliplatin and ActD (Fig. 2*B*). These nucleolar changes are accompanied by inhibition of rRNA transcription but not processing in oxaliplatin and ActD-treated cells. Radiolabeled 47S and 28S bands are both significantly reduced in intensity in RNA labeled after oxaliplatin and ActD treatments in comparison to cisplatin-treated and untreated controls (Fig. 2, *D* and *E*). The simultaneous reduction of both of these transcripts indicates that rRNA transcription, but not processing, is being affected by these drugs. A defect in RNA processing would be indicated by the reduction of the labeled 28S transcript, while 47S transcript levels would remain the same; an example is shown in Fig. S1 for the RNA processing inhibitor 5-fluorouracil (17). Quantification (Fig. 2*E*) shows that treatment with ActD almost completely eliminates both 47S pre-rRNA and the processed 28S transcripts. Following oxaliplatin treatment, 47S levels are reduced to less than 1% of the negative control and 28S levels reduced to 33%. This level of inhibition of transcription is similar to that previously observed with 6 μM oxaliplatin treatment in human fibrosarcoma cells (17). Cisplatin treatment, on the other hand, retained 60% of 47S transcript levels and 86% of 28S levels as compared with the negative control. Thus, both oxaliplatin and ActD robustly inhibit rRNA transcription but not processing, whereas cisplatin has a much lesser effect. It can be concluded with reasonable confidence that the relatively low level observed of rRNA transcription inhibition does not underlie cisplatin’s cytotoxicity, for reasons that will be explored later in this paper.

### Inhibition of rRNA transcription coincides with NPM1 redistribution upon oxaliplatin treatment

**Figure 3:**
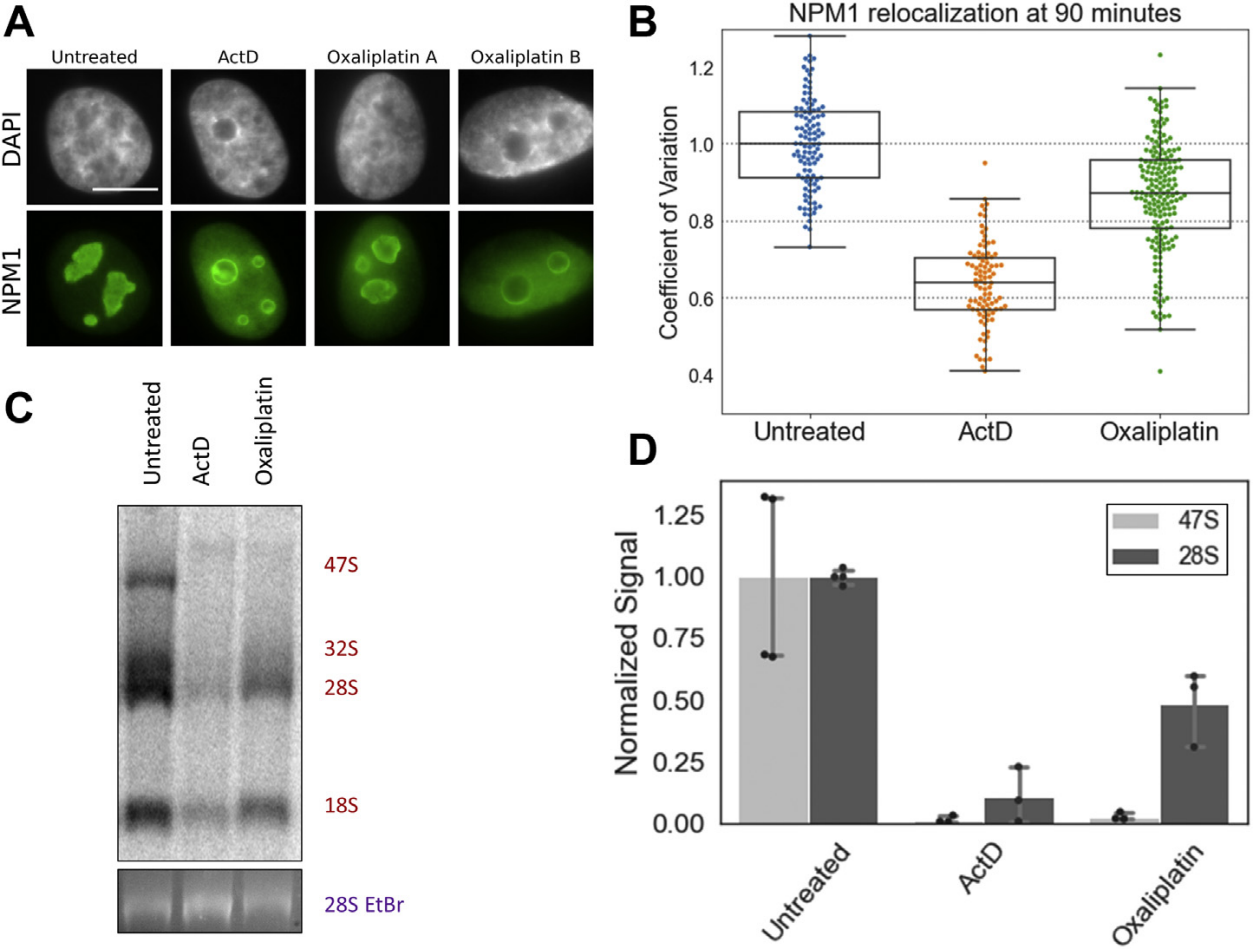
**NPM1 relocalization and inhibition of rRNA processing in A549 cells after 90 min of treatment with oxaliplatin.***A*, NPM1 relocalization after 90 min of treatment with 10 μM oxaliplatin or 5 nM ActD. Two examples of oxaliplatin-treated cells illustrate the intermediate state of nucleolar stress that the cells are in at 90 min of treatment, with a range of cells showing various levels of NPM1 redistribution and nucleolar rounding. *B*, quantification of NPM1 relocalization at 90 min of treatment with oxaliplatin and ActD. *C*, pulse-chase experiment after 90 min treatment with ActD or oxaliplatin. *D*, quantification of pulse-chase data from the gel image in 2D. Error bars represent the standard deviation of replicates separately treated in three wells of A549 cells across 2 days. Scale bar is 10 μm.

After establishing that rRNA transcription was significantly inhibited by oxaliplatin treatment, we wondered about the relationship between transcription inhibition and relocalization of NPM1. NPM1 is known to facilitate ribosome biogenesis, and depletion of NPM1 has been shown to inhibit pre-rRNA transcription (29). Thus, we considered that NPM1 relocalization may precede transcription inhibition. To examine this more closely, we tested both NPM1 relocalization and rRNA synthesis at an earlier treatment time point of 90 min. At 90 min, many cells clearly display the nucleolar stress phenotype in NPM1 images, but not to the extent that is seen at later time points (Fig. 3*A*). Many nuclei appear to be in an intermediate state in which NPM1 is partially translocated, with nucleoli that are not yet round (Fig. 3*A*, third panel). A few nuclei do appear to have completely entered a nucleolar stress state, with complete translocation of NPM1 and completely rounded nucleoli (example in Fig. 3*A*, fourth panel). This observation of intermediate levels of nucleolar stress following 90-min oxaliplatin treatment is supported by the median CV value of around 0.9 (Fig. 3*B*). Populations of cells undergoing extensive NPM1 translocation usually have median CV values below 0.7, as observed for the positive control of ActD and longer oxaliplatin treatment times (Figs. 1 and 2). Transcriptional inhibition is clearly occurring at this time point after oxaliplatin treatment as observed by the reduction of transcript levels (Fig. 3, *C* and *D*). Oxaliplatin shows a reduction of primary 47S transcript levels to about 3% of the control and 28S transcripts to under 50% of control levels. Thus, at 90 min of oxaliplatin treatment, 97% loss of primary 47S rRNA transcripts is observed through Pol I inhibition. At this same time, NPM1 redistribution is somewhat less extensive, with an intermediate CV value reflecting a range of individual cell morphologies. These observations suggest that Pol I inhibition precedes robust NPM1 redistribution or at least is contemporaneous with it.

### Pol I inhibition and DNA damage

**Figure 4:**
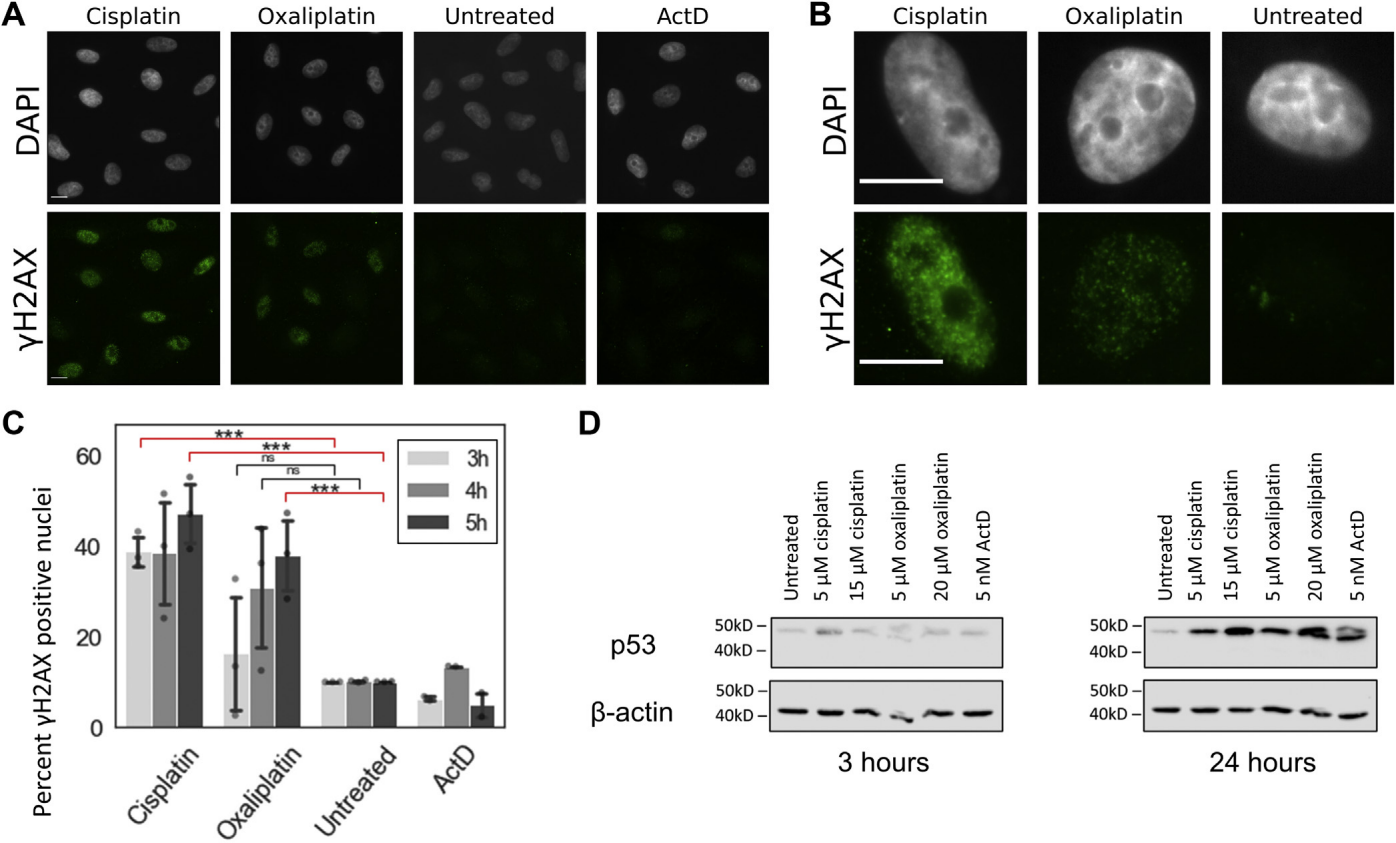
**DNA damage and p53 stabilization after treatment with platinum(II) compounds.***A*, DNA damage, measured by γH2AX immunofluorescence (*green*) after 5 h of treatment with cisplatin and oxaliplatin. *B*, representative single cell images of A549 cells after treatment with cisplatin and oxaliplatin. *C*, percentage of γH2AX positive nuclei, where a positive threshold was determined by the 90th percentile intensity of the untreated control for each testing day. Imaging was conducted on three separate days, with the exception of ActD, which was only tested on 2 days. Each point represents the percent positive nuclei for a single day and testing condition. *D*, western blot to detect p53 and loading control β-actin after Pt(II) treatment at 3 h (*left*) and 24 h (*right*). All scale bars are 10 μm. ∗∗∗*p* < 0.001, ns = *p* > 0.1.

It has been previously demonstrated that DNA damage can lead to the redistribution of NPM1 from the nucleolus to the nucleoplasm (26, 30) and that Pol I inhibition can result from damage to ribosomal DNA (23, 31, 32). Pt(II) compounds are well known to form cross-links and elicit a DDR in cells (33). Therefore, one possible mechanism by which oxaliplatin treatment results in NPM1 redistribution and Pol I inhibition is as a secondary response to DNA damage. If oxaliplatin-induced nucleolar stress was caused by underlying DNA damage, we would expect excessive or rDNA-localized DNA damage induced by oxaliplatin relative to cisplatin. Previous work has shown that oxaliplatin forms fewer DNA lesions than cisplatin by 2- to 6-fold after 4 h of treatment in A2780 and CEM cells as measured by atomic absorption spectroscopy (34, 35), and Bruno *et al.* observed γH2AX foci in cisplatin but not oxaliplatin-treated cells (14). These prior observations do not support general excessive oxaliplatin-induced DNA damage. We further tested possible correlations between the observed nucleolar and ribosome biogenesis stress responses to oxaliplatin and oxaliplatin-induced DNA damage. We completed a treatment time course with cisplatin and oxaliplatin from 3 to 5 h and performed immunostaining with both NPM1 and γH2AX. γH2AX is a phosphorylated histone variant resulting from activation of the DDR signaling pathway ATM and is a well-established marker that labels sites of DNA damage in cells (14, 23, 36), including nucleoli (37) (Fig. 4, *A* and *B*).

Nucleus-wide γH2AX staining, concentrated in foci, was observed upon cisplatin treatment as expected (Fig. 4, *A* and *B*). Contrary to what was previously described (14), we did observe some γH2AX staining with oxaliplatin treatment, although qualitatively this intensity appeared to be less than observed upon cisplatin treatment. There were very low levels of foci observed in the no-treatment control and with ActD treatment. NPM1 redistribution progressed as expected in both treatment conditions, with oxaliplatin displaying more extensive NPM1 relocalization than cisplatin at all three time points (Fig. S2). Using data from three separate testing days, differences in NPM1 redistribution between oxaliplatin and the untreated control were confirmed to be statistically significant after 4 h of treatment, while there was no statistically significant difference between cisplatin and the untreated control (Fig. S3).

Two approaches were used to quantify γH2AX as a function of treatment condition (Methods). The intensities of γH2AX staining per nucleus were quantified (Fig. S5*A*) as well as the intensities of individual foci (Fig. S5*B*). Cisplatin treatment yielded more cells with high total nuclear γH2AX (Fig. S5*A*) and more foci with higher intensity than other treatment conditions (Fig. S5*B*). Using the overall nuclear intensities, a “percent positive” value was calculated for each treatment condition based on a threshold of 90% of the control sample intensities. Nuclei in the experimental samples with integrated intensity levels higher than this threshold were counted as positive for γH2AX (Fig. 4*C*). A higher percentage of nuclei were positive in cisplatin-treated samples than under oxaliplatin conditions. The number of γH2AX-positive nuclei increased between 3 and 5 h with oxaliplatin treatment (Fig. 4*C*). In conclusion, while oxaliplatin-induced DNA damage is detected in these experiments, the results indicate that cisplatin is inducing a more robust DDR than oxaliplatin in particular at earlier time points. Further, Pol I inhibition, observed at 90 min treatment (Fig. 3), precedes any notable increase in γH2AX staining in oxaliplatin-treated cells. It is therefore unlikely that oxaliplatin’s strong nucleolar stress response is being caused by an upstream DDR.

In addition to nucleus-wide rRNA transcriptional silencing that can result from DNA damage (32), a localized inhibition of Pol I within individual nucleoli can occur after damage to rDNA, marked by γH2AX (38). Other research has shown that upon targeted rDNA damage, nucleoli reorganize and form nucleolar caps, which contain γH2AX foci (37). Among cells where nucleoli were clearly visible, this signature γH2AX localization in nucleolar caps was not observed in any of the treatment conditions (Fig. 4*B*, Fig. S4). Only a few cells displayed γH2AX foci near the nucleolus, and these did not resemble previously reported distinct perinucleolar foci (Fig. S4). Instead, foci were mostly observed to be distributed throughout the nucleoplasm, resulting in occasional proximity to or overlap with the nucleolus. This data suggests that oxaliplatin treatment is not resulting in γH2AX-detected damage to nucleolus-associated DNA. In sum, neither specific rDNA damage nor genome-wide DDRs are likely to be causing the observed nucleolar stress.

### Cisplatin and oxaliplatin both result in downstream p53 stabilization

Mammalian cell death upon activation of the nucleolar stress response is mediated by stabilization of the tumor suppressor protein p53 (3, 4, 27, 28). However, p53 can also be activated by a litany of other cellular stressors, including DNA damage (28). Previous work has shown a connection between inhibition of rRNA transcription and processing and p53 stabilization alongside changes in nucleolar proteins (6, 39). This connection had not been characterized previously with platinum compounds.

We examined p53 stabilization by measuring the increase in p53 levels by western blot (Fig. 4*D*). Both cisplatin and oxaliplatin treatments show stabilization of p53 in A549 cells by 24 h treatment time. At 3 h treatment, however, little p53 increase is observed with cisplatin, oxaliplatin, or ActD treatment. Thus, although these compounds result in different patterns of nucleolar changes at short time periods, they have similar patterns of p53 stabilization occurring by 24 h.

Previous work has shown that cisplatin has a 24-h IC-50 value of 12.8 μM in A549 cells, while oxaliplatin has a much higher 24-h IC-50 value of 81.5 μM (15). Therefore, with 3 h treatment at 10 μM concentrations for both compounds, oxaliplatin causes robust nucleolar stress and Pol I inhibition at just 12% of the 24-h IC-50 value, whereas cisplatin only displays minimal nucleolar stress at 78% of the 24-h IC-50 value. Both compounds cause robust p53 stabilization after 24 h of treatment.

### Nucleolar integrity following platinum treatment

**Figure 5:**
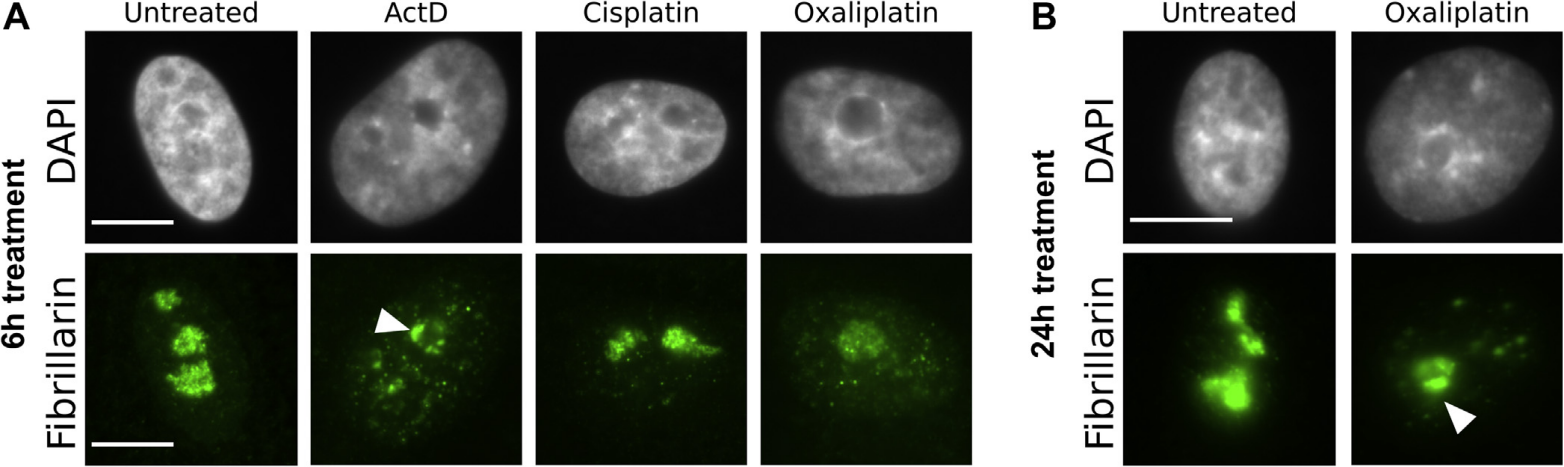
**Fibrillarin imaging after treatment with platinum compounds.** Representative images of fibrillarin immunostaining after 10 μM treatment with Pt(II) compounds or 5 nM ActD for (*A*) 6 h or (*B*) 24 h. Nucleolar caps indicated by *white arrow*. All scale bars are 10 μm.

To further examine the properties of nucleolar structure affected by treatment with platinum compounds, we used immunofluorescence to observe the localization of fibrillarin, another critical nucleolar protein (Fig. 5). Fibrillarin normally resides in the nucleolus in the dense fibrillar component. Fibrillarin-containing nucleolar caps form under conditions of nucleolar stress such as rDNA damage and inhibition of Pol I transcription (31). Previous work has shown that both cisplatin and oxaliplatin result in the formation of fibrillarin nucleolar caps at doses known to inhibit Pol I (17), which were, as described above, much higher for cisplatin (100 μM) than oxaliplatin (6.25 μM) in that study.

Interestingly, while cap-like structures were observed *via* NPM1 staining after just 3 h of oxaliplatin treatment (Fig. 2) and were continually observed up to 12 h after treatment, fibrillarin-containing nucleolar caps were not clearly detected after 6 h of oxaliplatin treatment when observing fibrillarin immunofluorescence (Fig. 5*A*). Fibrillarin-containing nucleolar caps were clearly observed as expected with the positive control of ActD (Fig. 5*A*). With both oxaliplatin and ActD treatments, nucleoli became rounder and more fibrillarin was detected in the nucleoplasm in comparison with the negative control. Fibrillarin distribution in cisplatin-treated cells largely resembled the negative control, with punctate foci observed in the nucleolus. Fibrillarin nucleolar caps do appear in oxaliplatin-treated cells after 24 h of treatment (Fig. 5*B*, Fig. S6). Thus, nucleolar integrity is being altered after 3 h of oxaliplatin treatment, but clear formation of fibrillarin-containing nucleolar caps does not occur until later time points. The timing of fibrillarin nucleolar cap formation after oxaliplatin treatment suggests that the relocalization of NPM1 may play an earlier role in nucleolar disruption than fibrillarin.

In sum, the imaging data from fibrillarin confirms again that the nucleolus is more affected by oxaliplatin than cisplatin under similar conditions. It also suggests that NPM1 translocation is not occurring independently as a unique phenomenon separate from the behavior of other nucleolar proteins, but as part of a global change to nucleolar structure and one that precedes fibrillarin localization into nucleolar caps.

## Discussion

The goal of this work was to better clarify the effects of different Pt(II) compounds on nucleolar stress-related processes. While both oxaliplatin and cisplatin have been reported to influence nucleolar processes, these effects occur at relatively higher cisplatin treatment concentrations (17). A recent report provides evidence that oxaliplatin causes cell death through a nucleolar stress response rather than through the DNA damage pathways long considered operative for cisplatin (14). This leads to a growing picture that while both compounds form lesions on DNA as well as other biomolecules, oxaliplatin acts primarily through an impact on the nucleolus. To further investigate how oxaliplatin impacts nucleolar processes and clarify its differences from cisplatin, we set out to directly compare the effects of these drugs on underlying biological processes that are associated with nucleolar stress. We focused on early treatment time points and examined redistribution of NPM1, performed pulse-chase assays to assess rRNA transcription and processing, observed DNA damage *via* γH2AX imaging, and further observed changes in nucleolar structure by imaging fibrillarin. These studies were performed in A549 cells, which are well characterized with respect to nucleolar stress pathways (40, 41).

At equivalent doses, oxaliplatin, but not cisplatin, was found to significantly inhibit rRNA transcription and not processing (Fig. 2). This inhibition happens after just 90 min of oxaliplatin treatment and is correlated with early translocation of NPM1 including formation of NPM1-associated nucleolar cap-like structures (Fig. 3). DNA damage is more extensive for cisplatin-treated cells, and in oxaliplatin-treated cells, DNA damage as measured by γH2AX is not observed extensively around nucleoli, suggesting that it is not a source of Pol I inhibition (Fig. 4). Although fibrillarin is redistributed from the nucleolus by 6 h of treatment with oxaliplatin, fibrillarin-containing nucleolar caps are not observed until later time points (Fig. 5), well after robust redistribution of NPM1 is observed. Thus, oxaliplatin at low doses causes global perturbation of the nucleolus, accompanied by Pol I inhibition and likely not driven by DNA damage. Cisplatin does not cause these nucleolar changes under similar treatment conditions. Cisplatin does cause more robust γH2AX appearance, consistent with a significant DDR. Taken together, our data support a model in which the DDR is a predominant mechanism of cell death upon cisplatin treatment (14). The data further indicate that prior observations of nucleolar responses at high cisplatin treatment concentrations (17, 19) are due to processes downstream of a primary DDR.

Both cisplatin and oxaliplatin lead to p53 stabilization in A549 cells by 24 h of treatment, but neither compound shows p53 stabilization at the earlier time points. Both rRNA transcription inhibition caused by oxaliplatin and DDR with cisplatin may cause downstream p53 stabilization by different pathways that have been established for nucleolar stress (17, 28) and for DDR (36, 42) in A549 cells.

In previous work, after establishing that oxaliplatin has a more similar sensitivity and resistance profile to ribosome biogenesis inhibitors than to DNA damage agents, Bruno *et al.* found that knockdown of multiple proteins involved in DDRs, including XRCC2, XRCC3, BRCA2, and FANC proteins, sensitized cells to cisplatin and carboplatin but not oxaliplatin. They also identified two checkpoint kinases involved in the DDR, Chk1 and Chk2, as critical to the sensitivity and resistance profiles of cisplatin but not oxaliplatin. Chk2 downregulation in particular was found to be a significant contributor to resistance to cisplatin but has much less of an effect on resistance to oxaliplatin. Further, they found that responsiveness to cisplatin in lymphoma cells injected into mice is affected by silencing Chk2. Specifically, silencing of Chk2 conferred resistance to these injected cells. This was not the case for oxaliplatin, with Chk2 having no effect on cell viability after injection. Further examination of the DDR in that study, combined with our observations of γH2AX in this paper, significantly supports that at clinically relevant doses, cisplatin-induced cell death is mediated by the DDR while the DDR is of minimal relevance to cell death upon oxaliplatin treatment in comparison with induction of ribosome biogenesis stress and more specifically Pol I inhibition.

Importantly, these observations open the possibility that oxaliplatin is a specific/selective RNA polymerase I inhibitor, in a class similar to small molecules such as BMH-21. Further studies might focus on which existing Pol I inhibitors oxaliplatin most closely resembles regarding the cellular behaviors it elicits. One proposed model for cisplatin’s previously described apparent ability to interfere with Pol I transcription is that the Pt(II) lesions it forms on DNA increase the binding affinity of transcription factors such as UBF and consequently “distract” these transcription factors from their role in facilitating rRNA synthesis and decrease transcription of rRNA (4, 43). Interestingly, however, this effect is limited to the cisplatin DNA lesions, and lesions formed by the oxaliplatin DACH ligand do not yield a higher UBF-binding affinity to rDNA (44). Additional work examined the effect of these ligands on the DNA-binding capacity of TATA-binding protein and High-Mobility Group Box proteins (HMGBs). Both HMGBs and UBF are in the HMG box family of DNA-binding proteins. For all proteins tested, the cisplatin ligand adduct on DNA was found to yield higher protein binding than the DACH lesion that would be generated by oxaliplatin, whereas the DACH ligand adduct did not have this effect (45). Given this data, if the UBF decoy model was correct, we would expect to see *more* inhibition of Pol I transcription with cisplatin treatment than oxaliplatin, as cisplatin lesions are more effective decoys than oxaliplatin lesions. Therefore, this model is contradicted by the now strong evidence that oxaliplatin is a stronger inhibitor of ribosome biogenesis than cisplatin (14).

Many questions remain with regard to platinum compounds and their relationship to nucleolar stress. For example, how does oxaliplatin inhibit RNA Pol I? To explore possible explanations, we can consider the mechanism of other Pol I inhibitors. For example, BMH-21 is known to cause stalling of rRNA transcription and ensuing degradation of Pol I subunits. Oxaliplatin may cause stalling of transcription or may prevent formation of the Pol I transcription complex. Stalling itself can occur in a number of ways, for example, oxaliplatin might bind to and alter RNA or DNA structures that modulate transcription. In addition to specific questions remaining about the relationship of particular chemotherapeutic drugs to nucleolar stress, overarching questions about the reorganization of the nucleolus remain. For example, what are the molecular steps governing nucleolar rearrangement following inhibition of rRNA transcription? NPM1 redistribution should be considered in a broader context of nucleolar disorganization, and it is noteworthy that this phenomenon appears to precede the formation of fibrillarin-containing nucleolar caps. The previously undescribed observation of NPM1 “cap-like” structures prior to the canonical caps formed with fibrillarin merits further investigation.

There may be a connection between this specific inhibition of Pol I by oxaliplatin and other processes that have shown differences between these platinum compounds. For example, immunogenic cell death has been shown to be more affected by oxaliplatin, and damage to the neurons *via* disruption of the neuronal nucleolus also appears more affected by oxaliplatin than cisplatin based on existing literature (46, 47), an interesting phenomenon when considering that oxaliplatin disproportionately results in peripheral neuropathy in patients (12). The questions and connections above are critical to a better understanding of the mechanisms of platinum-induced nucleolar stress and possible specific inhibition of Pol I by oxaliplatin. Exploration of this topic might lay the groundwork for a better clinical understanding of these drugs.

## Experimental procedures

### Cell culture and treatment

A549 human lung carcinoma cells (#CCL-185, American Type Culture Collection) were cultured at 37 °C, 5% CO_2_ in Dulbecco's Modified Eagle Medium (DMEM) supplemented with 10% Fetal Bovine Serum (FBS) and 1% antibiotic-antimycotic. Treatments were conducted on cells that had been grown for 11 to 25 passages to 70% confluency. Platinum compound treatments were conducted at 10 μM, and Actinomycin D treatments were conducted at 5 nM. Platinum compounds were made into 5 mM stocks on the day of treatment in 0.9% NaCl (cisplatin) or water (oxaliplatin). Stock solutions were diluted into media immediately prior to drug treatment.

### Immunofluorescence

Cells to be imaged were grown on coverslips (Ted Pella product no 260368, Round glass coverslips, 10 mm diam, 0.16–0.19 mm thick) as described above. After treatment, cells were washed twice with phosphate buffered saline (PBS) and fixed for 20 min at room temperature in 4% paraformaldehyde in PBS. PFA was removed *via* aspiration, and cells were then permeabilized with 0.5% Triton-X in PBS for 20 min at room temperature. Two 10-min blocking steps were performed with 1% bovine serum albumin (BSA) in PBST (PBS with 0.1% Tween-20). Cells were incubated for 1 h in primary antibody (NPM1 Monoclonal Antibody, FC-61991, from Thermo Fisher, 1:200 dilution in PBST with 1% BSA; anti-Fibrillarin antibody ab4566 from Abcam, 1:500 dilution; anti-Phospho-Histone H2A.X, 14-9865-82, from Thermofisher, 5 μg/ml) and 1 h in secondary antibody (Goat Anti-Mouse IgG H&L Alexa Fluor 488, ab150113, Abcam, 1:1000 dilution in PBST with 1% BSA), with three 5-min wash steps using PBST between incubations, and were washed in the same manner again before mounting slides. Coverslips were mounted on slides with ProLong Diamond Antifade Mountant with DAPI (Thermo Fisher) according to manufacturer’s instructions.

### Pulse-chase

A549 cells were grown to 70% confluency in 6-well tissue culture plates as described in Cell culture and treatment section above. Cells were then treated for the indicated amount of time in DMEM supplemented with 10% FBS and 1X antibiotic-antimycotic with the drug of interest added. Prior to the pulse step, phosphate depletion was performed for 1 h by switching regular media for phosphate-free media (including the previously mentioned FBS and antibiotic-antimycotic), which still contained the drug of interest. For the pulse step, media was replaced with a solution of 15 μCi/ml ^32^P orthophosphate made in phosphate-free media (with FBS and antibiotic-antimycotic). After a 1-h pulse step, media was replaced with cold drug-containing DMEM as in the first step of treatment for a 3-h chase step. After this chase step, RNA was extracted from the cell using a Zymo Quick-RNA Miniprep kit, separated by size on an agarose gel, and then visualized. The gels were visualized in two ways: 1) Total RNA that was visualized with an ethidium bromide stain and 2) only the radioactively labeled RNA that was produced during the pulse step was visualized. To achieve this, the gel was dried on Whatman paper using a gel dryer set for 2 h at 70 °C, after which it was left on the gel dryer overnight at room temperature. The dried gel was exposed to a phosphor screen for 24 h, and the screen was imaged using a Storm phosphorimager. The amount of labeled RNA was quantified by calculating the intensity of the gel bands in the images in ImageJ (48). Prior to quantification, .gel files from the Storm software were converted from square root encoding to linear encoding using the Linearize GelData ImageJ plugin (https://imagej.nih.gov/ij/plugins/linearize-gel-data.html). The amount of radiolabeled RNA was normalized to the total RNA levels for each lane as measured by EtBr. The amounts of the different RNA transcripts are shown in the graph as a fraction of the mean untreated control intensities for each experiment.

### Western blotting

Cells were removed from tissue culture plates *via* trypsinization and subsequently lysed with RIPA buffer. Blocking step was performed in 5% milk in PBST at room temperature from 1 h to overnight. Blot was incubated at room temperature for 1 h in primary antibody (1:500 p53 Monoclonal Antibody, MA5-12557, from Thermo Fisher or 1:1000 beta-Actin Monoclonal Antibody, AC-15, from Thermo Fisher, both in 5% milk in PBST) and 1 h in secondary antibody (1:10,000 Goat anti-Mouse IgG (H + L) Cross-Adsorbed Secondary Antibody, HRP, from Thermo Fisher). The blot was stripped and reprobed for each antibody. Chemiluminescence was performed with SuperSignal West Pico Plus kit, and the blot was rinsed three times for 1 to 5 min in PBST between incubation steps. The blot was imaged with Li-Cor imaging system and software.

### Imaging and quantification

Images were taken using a HC PL Fluotar 63×/1.3 oil objective mounted on a Leica DMi8 fluorescence microscope with Leica Application Suite X software. Quantification of NPM1 relocalization was performed (15) in an automated fashion using a Python 3 script. Images were preprocessed in ImageJ (48) to convert the DAPI and NPM1 channels into separate 16 bit grayscale images. A minimum of ∼80 cells were analyzed for each treatment group. Nuclei segmentation was determined with the DAPI images using Li thresholding functions in the Scikit-Image Python package (49). The CV for individual nuclei, defined as the standard deviation in pixel intensity divided by the mean pixel intensity, was calculated from the NPM1 images using the SciPy Python package. All data were normalized to the no-treatment control in each experiment. Data are represented as boxplots generated using Matplotlib and Seaborn within Python.

Quantification of γH2AX intensity and foci was performed with CellProfiler 3.0 software (50). In one analysis method, a “percent positive” value was calculated for each treatment condition relative to the untreated control. A threshold was determined for a positive γH2AX result based on the 90th percentile intensity value of the untreated control for each time point. Nuclei in the experimental samples with integrated intensity levels higher than this were counted as positive for γH2AX (Fig. 4*C*). Significance testing was conducted *via* two-sided *t*-test using the SciPy stats package. Histograms showing integrated intensity/nucleus for all nuclei are shown in Fig. S5*A*. In addition to integrated γH2AX intensities, the intensities of individual foci were also quantified (Fig. S5*B*).

## Data availability

The data that support the findings of this study are included within the article and its supporting information.

## Supporting information

This article contains supporting information.

## Conflicts of interest

The authors declare that they have no conflicts of interest with the contents of this article.
