# Supplementary material for: Early nucleolar responses differentiate mechanisms of cell death induced by oxaliplatin and cisplatin
Source: J Biol Chem. 2021 Apr 3;296:100633. doi: 10.1016/j.jbc.2021.100633 (PMC8131322; doi:10.1016/j.jbc.2021.100633)
Supplement: Supplemental Figures S1–S6 [file mmc1.pdf]

Supporting information for:

Early nucleolar responses differentiate mechanisms of cell death induced by oxaliplatin and cisplatin

Emily C. Sutton[1,2], Victoria J. DeRose[2,3,*]

[1]Department of Biology, University of Oregon, Eugene, OR

[2]Institute of Molecular Biology, University of Oregon, Eugene, OR

[3]Department of Chemistry and Biochemistry, University of Oregon, Eugene, OR

*Corresponding author: Dr. Victoria DeRose

Email: derose@uoregon.edu

Running title: Nucleolar responses to oxaliplatin and cisplatin

Supporting Information

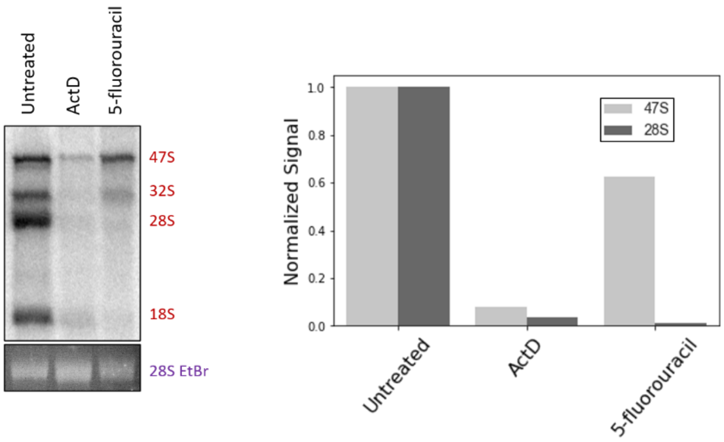

***Figure S1** – Positive controls for pulse chase assay*. Left panel shows pulse chase data resulting from sample control conditions. The negative control is RNA from untreated A549 cells, which show labeled 47S, 32S, 28S, and 18S rRNA transcripts. The positive control for rRNA transcription inhibition, 5 nM Actinomycin D, demonstrates a significant reduction in all rRNA transcripts. The positive control for rRNA processing inhibition, 200 µM 5-fluorouracil, shows a significant reduction in the 32S, 28S, and 18S rRNAs, while the 47S band remains prominent. Cells were treated for 3 hours prior to the pulse step. The right panel shows quantification of this gel data, with radiolabeled bands being normalized to total RNA as measured by EtBr, and further normalized to the untreated control. Quantification confirms a large reduction in the both 47S and 28S rRNA after Actinomycin D treatment, while 5-fluorouracil causes a significant reduction in the processed 28S transcript, with only a modest reduction in the primary 47S transcript.

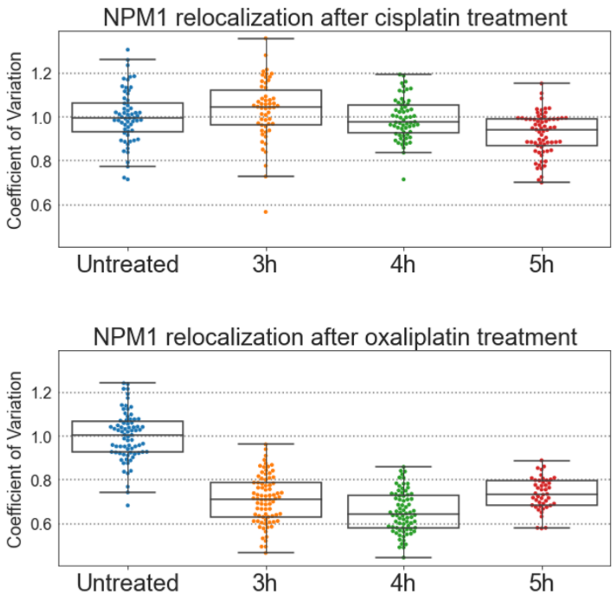

***Figure S2** – NPM1 redistribution at time points observed for γH2AX.* Quantification of NPM1 redistribution between 3-5 hours of treatment with cisplatin (top) and oxaliplatin (bottom). NPM1 redistribution was observed simultaneously at times when γH2AX was being measured. Data above represents a single day of testing in A549 cells. Treatments were completed with 10 µM of each compound.

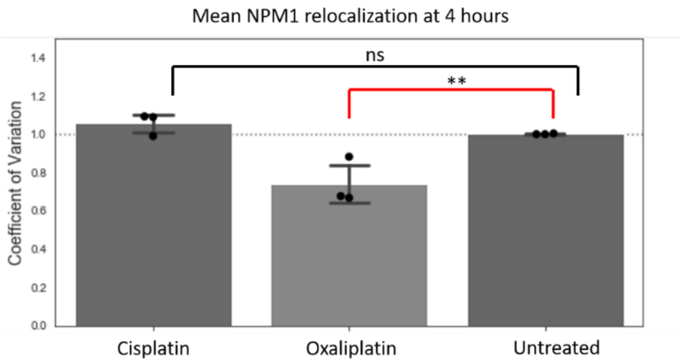

Figure S3 – *Difference in NPM1 relocalization at 4h treatment*. Each point on the graph is a mean, normalized CV for an individual testing day, with a lower mean CV representing more NPM1 redistribution. This chart represents three trials over three separate days. A two-sided t-test was performed with the SciPy stats package using the mean CVs for each day. A statistically significant difference was found between the untreated samples and oxaliplatin, but not between untreated and cisplatin. ** = $p<0.05$, ns = $p>0.1$

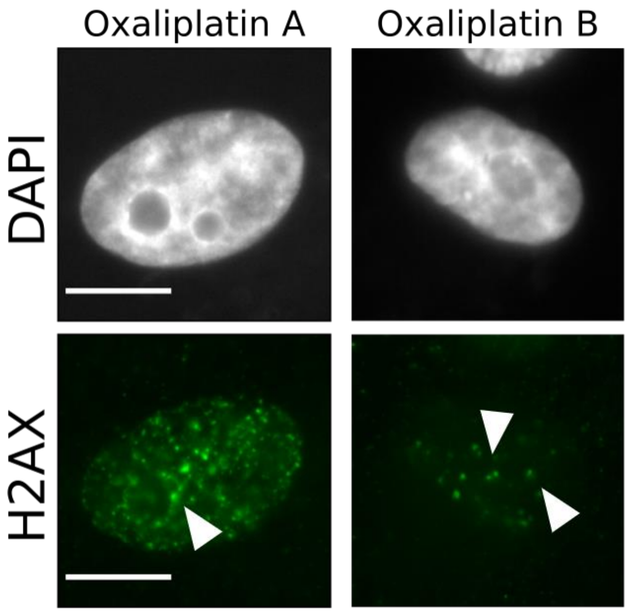

Figure S4 – *γH2AX foci around the periphery of the nucleolus in oxaliplatin-treated cells.* γH2AX foci have been previously reported to form in nucleolar caps after damage of ribosomal DNA. A small number (less than 1 in 40) of oxaliplatin treated nuclei showed γH2AX foci around the periphery of the nucleolus, demonstrated in this figure. These foci did not resemble distinct perinucleolar foci that would indicate rDNA damage. The low number of cells displaying nucleolar-adjacent γH2AX foci suggests that rDNA damage is likely not driving oxaliplatin-induced nucleolar reorganization. Scale bar 10 µm. Treatment was completed with 10 µM oxaliplatin.

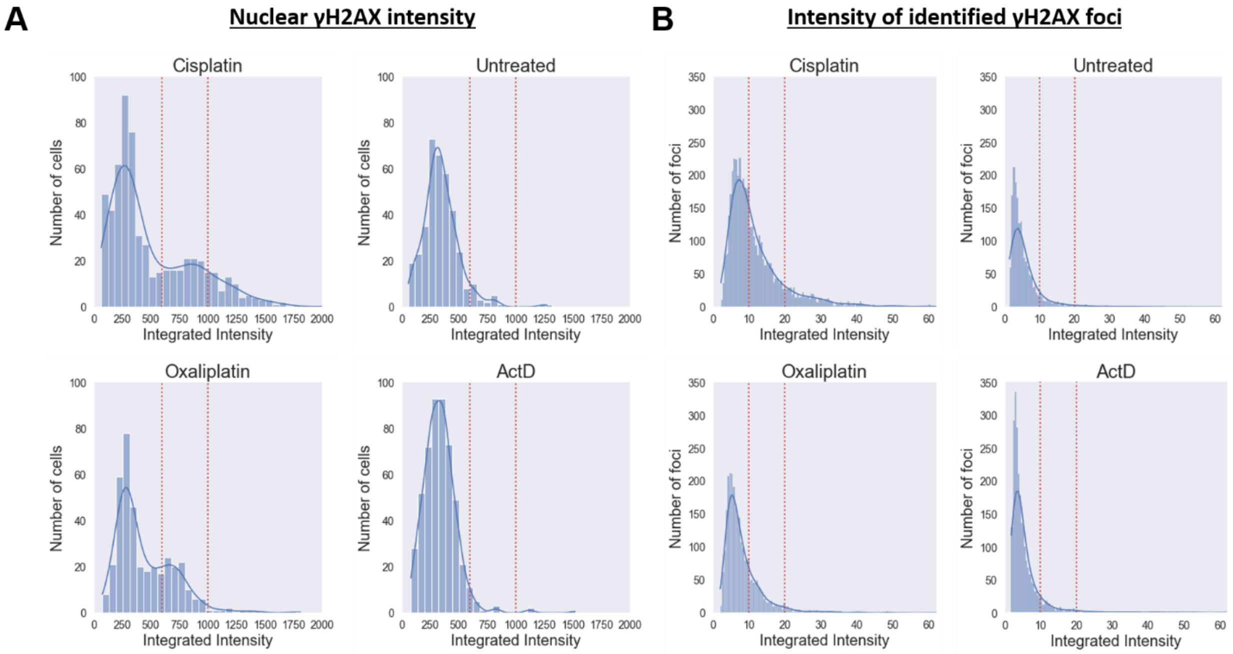

Figure S5 – *Quantification of γH2AX nuclear intensity and foci intensity in A549 cells.* A) Distribution plots of nuclear γH2AX intensity. Y-axis represents integrated intensity of γH2AX within a segmented nucleus, X-axis represents the number of nuclei with a given intensity. Unlike the untreated and ActD treated samples, both cisplatin and oxaliplatin have a population of nuclei with higher total γH2AX intensity. However, the distribution plot for cisplatin skews farther to the right than oxaliplatin, indicating that γH2AX stained cells are brighter (confirming qualitative observations) in cisplatin treated cells than oxaliplatin treated cells. B) Distribution of intensities of all γH2AX foci identified in each population of cells. The X-axis represents the integrated intensity of a single γH2AX focus, and the Y-axis represents the number of foci at a given intensity. More foci of high intensity (brighter foci) were identified in cisplatin treated cells than all other samples. Regions of interest where differences between the samples can best be observed are demarcated by red dotted lines. The data in this figure represents one day of testing at 3 hours of treatment in A549 cells. Treatments were done at 10 µM for Pt(II) compounds and 5 nM for ActD.

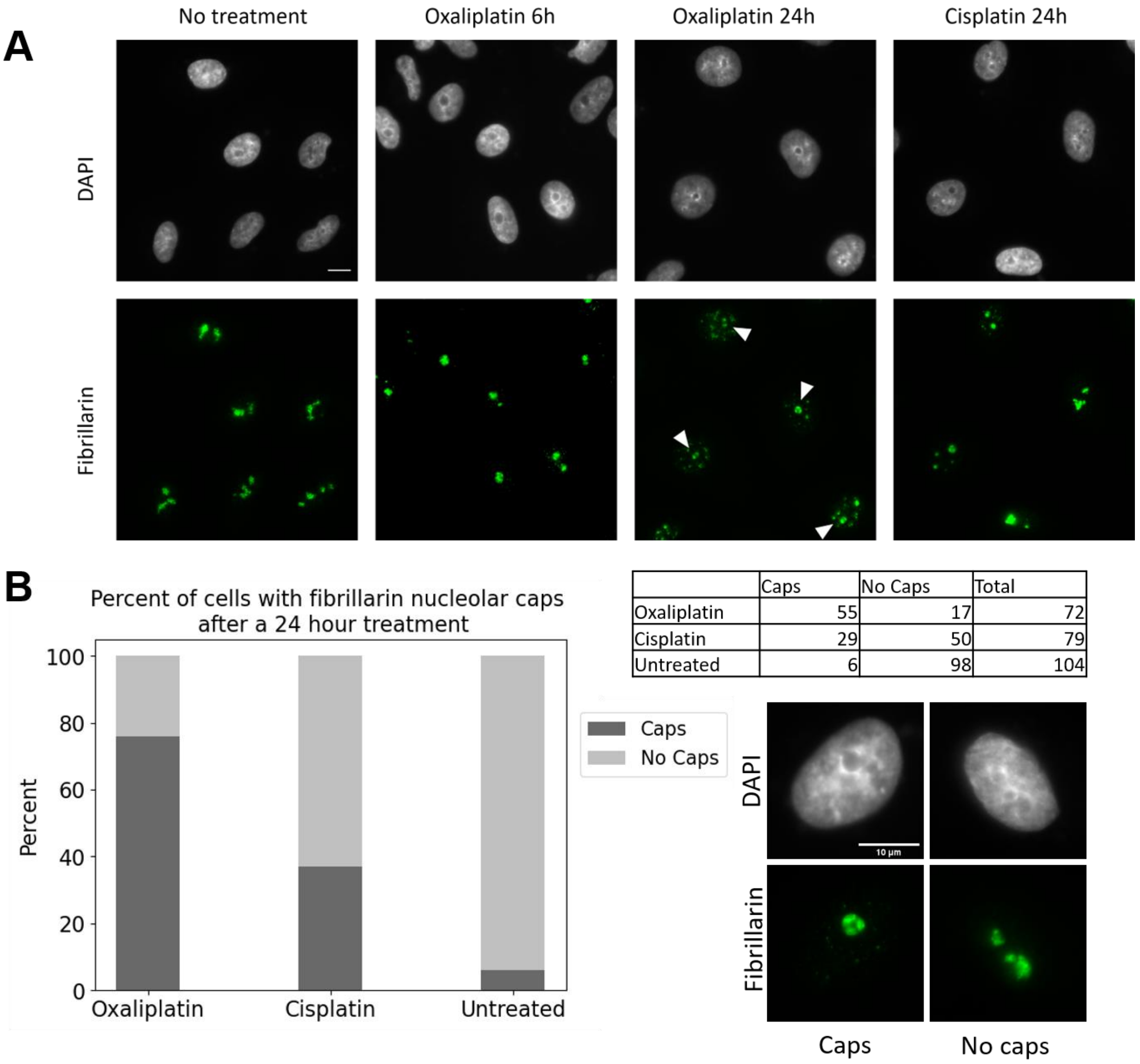

	Caps	No Caps	Total
Oxaliplatin	55	17	72
Cisplatin	29	50	79
Untreated	6	98	104

Figure S6 *– Fibrillarin staining at 6 and 24 hours of treatment with oxaliplatin and cisplatin.* A) Nucleolar caps are not observed at 6 hours of treatment with oxaliplatin, but are observed at 24 hours of treatment (indicated by white arrows). Nucleoli are rounded at 6 hours, as observed by both DAPI and fibrillarin staining, suggesting that nucleolar stress is occurring despite the lack of nucleolar caps at this time. At 24 hours of cisplatin treatment, some nucleolar caps have formed, but most cells resemble the 6 hour oxaliplatin treatment without prominent caps. Treatments were done at 10 µM oxaliplatin and cisplatin. B) Quantification of cells with fibrillarin nucleolar caps. Cells across five images for each treatment condition were scored, with each cell being manually classified as either containing nucleolar caps or not containing nucleolar caps. Counts are shown to the right of the stacked bar chart representing this data. Only 6% of cells in the untreated sample had caps, which were found in a majority of oxaliplatin treated cells (76%), and in only 37% of cisplatin treated cells. Below the chart is an example of cells with caps, from the 24 hour oxaliplatin treatment, and cells without caps, from the 24 hour cisplatin treatment. Scale bars are 10 µm.
